# Traditional medicine and global health: a call for papers 

**DOI:** 10.2471/BLT.24.292621

**Published:** 2024-11-01

**Authors:** Shyama Kuruvilla, Geetha Krishnan Gopalakrishna Pillai, Tanja Kuchenmüller, Susan Wieland, Bhushan Patwardhan, John Reeder

**Affiliations:** a Global Traditional Medicine Centre, World Health Organization, ITRA campus, Jamnagar, Gujarat 361008, India.; b Research for Health, World Health Organization, Geneva, Switzerland.; c Cochrane Complementary Medicine, University of Maryland School of Medicine, Baltimore, United States of America.; d Interdisciplinary School of Health Science, Savitribai Phule Pune University, Maharashtra, India.

Traditional medicine, including traditional, complementary, integrative, Indigenous and ancestral practices, is a vital resource for billions of people worldwide, serving as either primary access or preferred choice for health and well-being needs. These practices provide unique perspectives on health and well-being and, in some cases, incorporate contemporary science. 

Today we face pervasive disequilibrium – with socioeconomic disparities, political instability, gender inequalities, violence and rapid technological change. By seeking to restore balance for individuals, societies and environments, traditional medicine aligns with frameworks around equity, One Health, planetary health and the United Nations sustainable development goals (SDGs).^1^

Demand for traditional medicine is growing, as illustrated by the estimated increase of the wellness economy associated with traditional medicine: in 2022 it was valued at 5.6 trillion United States dollars (US$), and it is projected to reach US$ 8.5 trillion by 2027.^2^ To ensure the accessibility, safety, efficacy and sustainability of the use of traditional medicine, more robust scientific evidence and regulatory frameworks are needed.^3^

Indigenous Peoples possess invaluable knowledge of traditional medicine. However, colonization and exploitation have threatened their ways of life. Realizing Indigenous rights is a shared global imperative, as is fostering respectful knowledge exchange with the scientific community. 

Approximately half of all approved contemporary drugs derive from natural products, which are also assessed to be the best source for novel therapeutic agents.^4^ A few thousand approved biomedical drugs exist,^4^ while hundreds of thousands of traditional medicine formulations, for example in India’s Traditional Knowledge Digital Library,^5^ remain scientifically underexplored. Concerns about related bioprospecting and inequities in access and benefit sharing are addressed in the World Intellectual Property Organization treaty on intellectual property, traditional knowledge and genetic sources.^6^

Traditional medicine can also contribute to universal health coverage (UHC). For example, in China, almost all hospitals practising Western medicine have departments of traditional Chinese medicine.^7^ In some parts of Africa, traditional healers are more numerous than conventional doctors.^8^ The United States Veterans Affairs Whole Health approach includes traditional medicine.^9^ Still, traditional medicine is not systematically considered in health policy and planning.

To harness the potential of traditional medicine, the global health community must prioritize evidence-based innovation. First, acknowledging that traditional medicine has a long history of contributing to scientific breakthroughs – from smallpox inoculations to aspirin from willow bark, contraceptive pills from yam, artemisinin for malaria and childhood cancer treatments derived from rosy periwinkle.^10^ While randomized controlled trials provide a robust standard of evidence, their use can be complex when evaluating traditional medicine due to its personalized, holistic approaches.^11^ Therefore, innovative research methods are needed such as practice-based evidence.^1^ Systems biology, pharmacogenomics and digital health applications, could help scientifically substantiate the safety and efficacy of some traditional medicine.

As we prepare for the second WHO Traditional Medicine Global Summit in November 2025,^12^ the *Bulletin of the World Health Organization* will be producing an accompanying theme issue that will explore the profound potential of traditional medicine. The *Bulletin* therefore calls for high-quality papers on the nexus of traditional medicine and modern science including: multidisciplinary research methods and evidence-informed decision-making; UHC and health systems; biodiversity, sustainability and the use of natural resources; Indigenous Peoples’ rights and respectful knowledge exchange; intellectual property rights and equitable benefit-sharing; digital health and technological advances; and the contribution of traditional medicine to global health priorities.

The deadline for submissions is 1 March 2025. Manuscripts should be submitted in accordance with the *Bulletin*’s guidelines for contributors and the cover letter should mention this call for papers.
